# Impact of COVID-19 and associated lockdown on livestock and poultry sectors in India

**DOI:** 10.14202/vetworld.2020.1928-1933

**Published:** 2020-09-21

**Authors:** Jyotsnarani Biswal, Kennady Vijayalakshmy, Habibar Rahman

**Affiliations:** International Livestock Research Institute South Asia Office, Pusa, New Delhi, India

**Keywords:** COVID-19, labor availability, livestock sector, poultry sector, value chain

## Abstract

The COVID-19 pandemic and the associated lockdown for a long period have created a significant adverse impact on different sectors, including that of the agriculture and other allied sub-sectors in India and several other countries. The present review aimed to depict the impact of this pandemic and the lockdown on the livestock and poultry sectors in the country, which has been one of the fastest-growing sectors in recent years. Inadequacy of country-wide information has been a major bottleneck for having a thorough understanding of the impact of the prolonged lockdown on different sub-sectors of livestock and poultry. In the present case, an in-depth analysis of the subject has been made through the collation of available published materials and information collected through public contacts. The pandemic and the associated lockdown has not only caused enormous distress to the millions of poor and marginal farmers for saving their crops and/or livestock and thereby assuring their livelihoods but also impacted the overall poultry, dairy, and other livestock production systems and associated value chains, nutrition and health care, and labor availability. The paper highlights various dimensions of the impacts, namely, reduction in demand of different commodities, wastage of the produce due to the closure of transport and market chains, distress sale of the produce, and labor shortage and revival strategies taken by the government and associated enterprises. The present impact study although gives a picture about the overall present scenario, a systematic study through the collection of primary data from all over the country is suggested, which will provide a holistic view of the impact on each of the sub-sectors and the associated value chains.

## Introduction

The ongoing coronavirus pandemic (COVID-19) has been a public health emergency all over the world, which further becomes more important for a developing and also populous country like India with a population of over 1.3 billion. The enforcement of three-phase continuous lockdown for such a long period of more than 1½ months has been a new experience for everyone. The distress of millions of poor and marginal farmers for saving their crops and/or livestock and thereby assuring their livelihoods is not only a concern of the government but also for all those associated with the sector directly or indirectly. Livestock and poultry have been important sub-sectors of agriculture in the country, contributing 4.9% (Rs. 758,417 crores in 2017-2018) of the totalgross value added of the country [1] and providing livelihood and employment to about 8.8% population in the country. With a population of 536.76 million livestock and 851.81 million poultry [2], the sector has been the major provider of animal protein for both non-vegetarians and vegetarians in the country, thereby significantly contributing to nutritional security of all age groups.

The impact of COVID-19 and the associated lockdown on livestock and poultry sectors in the country during this period has been phenomenal. It is further envisaged that the impact would continue to be long-standing and will have great bearing on the livelihood, employment, and overall economy of the sector. While all associated issues are being addressed with a strong might in these days, a holistic understanding of the overall impact would help in drawing appropriate policies and revival strategies.

Inadequacy of country-wide information on relevant aspects has been a major bottleneck for having a better understanding of the impact of the pandemic and the prolonged lockdown on different sub-sectors of livestock and poultry. An attempt, therefore, has been made to have an in-depth analysis of the subject through the collation of published materials and also information collected through public contacts, a summary of which is presented herewith.

## Impact on Dairy and Associated Value Chains

India, with milk production of 187.7 million tonnes (2018-2019), has been continued to occupy the crest position in the globe [3]. This has been growing over 6% annually since 2014-2015 [4]. With the incidence of COVID-19, the dairy industry in India has suffered significantly due to the reduced overall demand of about 25-30% in the country, at least during first 1 month after the lockdown, that is, since March 25, 2020 [5]. As per the available reports, with the immediate declaration of lockdown, a good number of consumers undertook the bulk purchase of milk to meet their requirements for about 5-7 days. This had led to a surge of 15-20% demand for milk during the initial 2 days, which, however, shown a drastic slow-down in subsequent days. With the closure of the roadside tea stalls, road-side eateries, restaurants, and hotels during the lockdown, a share of about 15% of total milk consumption in the country were almost at complete halt [5].

The distress sale of milk by the farmers, even up to 50% reduction in price, was reported to happen in several rural areas where there has not been the existence of any milk cooperatives or any private agencies for such marketing [6,7]. Non-conduct of any cultural ceremonies and professional events, which generally utilize the milk in bulk, further added to its reduced demand, although the quantification of the same may be difficult at this stage due to lack of adequate data. The sweet shops who had been the permanent customers for several dairy farmers are no more in a position to procure any milk or cheese, as their shops have been closed since past 1½ months, and further, they are not sure how long more they need to wait for initiating their business. With no other available markets, these farmers have been observed in milking their animals only once a day, thereby losing substantial money. Sealing of the borders by several states to prevent the vehicular movements and also the movement of the migrants, as a step toward control the spread of the COVID-19, further hindered the transportation of the milk to the consumers, at least in the border regions.

A published report in the print media shows that while the demand for several milk products, namely, ice-cream, milkshakes, and lassi, which are the sort-after items for the summer, reduced drastically; the demand of paneer, ghee, and cheese has been on a sharp rise in these days. Inadequate access to the other animal products such as chicken, meat, and fish has also subjected to higher demand for products such as paneer by the non-vegetarian population. The household consumption of ghee, butter, and milk found to increase day-by-day amid the lockdown period, as most people have been forced to stay at their home. However, the household consumption being the contributor of only about 25-30% of the overall milk usage of the country, such marginal increase in consumption was insufficient to compensate for its decline usage [7].

As per the Press Release by the National Dairy Development Board (NDDB) dated March 25, 2020, to counter the distressing scenario, NDDB had appealed to all dairy cooperatives across the country for making suitable arrangements for uninterrupted collection, chilling, transportation, and processing of milk and availability of fodder in villages, and also to take essential measures for a continued supply of milk and milk products to avoid situations of panic buying by the consumers [8]. As per the available reports, following the guidelines/instruction of the government, the dairy cooperatives were continued to procure the milk as before, and in certain cases much higher in quantity than the normal was procured, even though there was a substantial decrease in demand. Amul in Gujarat and Karnataka Milk Federation are a few examples of such cooperatives. In such cases of higher procurement, the surplus milk was used mainly for skimmed milk powder (SMP) production. It was pertinent to mention that the pre-lockdown period did experience a lull in demand for SMP in the domestic markets, registering a decline of about Rs. 80-90/kg, which was similar to that of international markets trend. Added to that, with the excess production of SMP, it is envisaged that the lower price of the SMP would continue to prevail in coming days too.

The smaller cooperatives in several states, namely, West Bengal, Odisha, and Jharkhand, on the other hand, could not be able to maintain the procurement levels. Further, in several cases, the procurement of milk was reported to happen with reduction of about Rs. 3-5/L in procurement price. The spread of the COVID-19 being comparatively higher in some major milk-producing states such as Gujarat, Maharashtra, Karnataka, and Tamil Nadu in the initial disease development phase, the issue of reduced demand has become more prominent.

Switching over from the fresh milk delivered at the doorstep to buying of packaged milk by several consumers was a great setback for the small-scale farmers in both urban and rural areas for the marketing of their milk. It is known that a cow, whether it produces milk or not, needs about 25 kg of fodder every day. The lack of adequate working capital and reduction of cash flow further were other bottlenecks for procurement of critical feed inputs and the fodder.

The demand-supply gap is found to decrease gradually with the opening-up of market chains and also additional steps taken by the local administrations in plugging the bottlenecks in delivery systems. The private players, in general, having higher product portfolios were found to be more active in supplying their products through the online platform and home delivery. The sale of the products through informal channels such as neighborhood marketing has been made successful by certain entrepreneurs. Several such actions subsequently led to increased household consumption, and the present level of demand is reported to be only 10-12% lower than the normal [9]. As per the press release of NDDB dated April 18, 2020, the percentage of liquid milk procurement during post-COVID-19 (March 16-April 16, 2020) period was declined only by 8.8% when compared with pre-COVID-19 (March 1-March 15, 2020) period [5].

## Impact on other Livestock Production and Value Chains

The setback observed in the piggery sector was quite significant, perhaps similar to that of dairy. Lack of marketing opportunity was probably the most important issue, which forced the farmer/owner for possession of the animal for a longer period, even much beyond the marketing age. Such action led to continued feeding of animals and increased feed conversion ratio, thereby heavy economic loss to the keepers. Longer rearing period causing over-growth of animals also resulted in the reduction of market acceptance and thus lower income. Limited access to the commercial pig feed due to restriction of transport was another major issue encountered by the commercial piggery in particular. When the country was already battling to overcome from the threat of COVID-19 outbreak, the State of Assam in the North-Eastern parts of India was gripped under the threat of African Swine Fever. The deadly viral disease reported killing over 14,000 pigs across 14 districts in Assam within 15 days of the outbreak [10]. The disease was also confirmed by the Indian Council of Agricultural Research (ICAR)-National Institute of High-Security Diseases, Bhopal. As an immediate measure to find the disease intensity and stopping its further spread, the ICAR-National Research Center on Pig at Rani, Assam, also initiated necessary research programs and provided advisories to the pig farmers. The lockdown was found to be beneficial due to restricted movements of the people and affected animals, thereby restricting the spread of the virus.

The goat farming practiced in the country being largely an extensive system, the investment in supplementary feeding is almost non-existent. Further, as the goat farmers are generally small/marginal farmers and landless laborers, who usually possess only 4-6 animals/family, the farming was probably least affected. However, as in other cases, the marketing of the animals remained a major constraint, thereby affecting the livelihood of these farmers who otherwise were depending on the sale of these animals. Similarly, the sheep rearing in the country is also an extensive farming activity, where the animal keepers are generally nomads, who leave home with their sheep flocks just after winter months in search of pasture and providing greater access for grazing their animals. In certain places, like in Jammu and Kashmir, the lockdown found to delay the spring migration of thousands of nomadic pastoral tribes, which may imply reduce food availability for their animals and ultimately impacting income.

The farmers possessing horses and ponies, which are used in good numbers in several religious places as a major mean of conveyance for the pilgrims in high hilly regions of Uttarakhand, Jammu and Kashmir, etc., and also for transport of goods, have almost lost their livelihood due to the total halt of the tourism. This is similar in case of some camel owners, especially in Rajasthan, who were depending on the desert tourism.

The domestic meat sector has also been severely affected, primarily due to the disruption of the market chain. Reduced availability of mutton, in particular, has shown skyrocketing its market price, with an increase of 50-80% of the normal price, in several cities of the country. On the contrary, with the countrywide lockdown, the buffalo meat export units had to close down their activities, which not only affected the associated stakeholders but also significantly affected the export earnings of the country, which has been over Rs. 25,000 crores per year.

## Impact on Poultry

India, at present, is the fourth-largest poultry producers in terms of volume. It is estimated that during the calendar year of 2019 about 3.8 million tonnes of poultry meat was consumed in the country, which is valued at about Rs. 85,000 crores in terms of retail price [11-13]. At the same time, the egg production of the country was estimated at 109 billion eggs, valued at about Rs. 45,000 crores. When continued growth performance of 10-12% in the sector, as witnessed in the past 3 years, was anticipated during the current year too, the incidence of COVID-19 pandemic at the beginning of the year brought an unprecedented impact on the sector. Unlike most of the economic sectors, the impact on the poultry sector in India was rather more pronounced even before the imposition of the country-wide lockdown. Even before the country registered the first case of COVID-19, the rumors of poultry birds as the likely carrier of the virus widely circulated in social media had led to reduced demand of the chicken meat in several parts of the country. Of late, the clarification issued by different agencies that eating chickens is quite safe, however, could able to convince the consumers to a great extent.

The post-COVID-19 lockdown further reduced the demand of the meat all across the country due to several other logistic factors. Even though the Union and State Governments did not have many restrictions on the opening of shops dealing with food commodities, including that of sale of meat and eggs, the reduced movement of people hampered the market of these products. Probably, most of the non-vegetarian population did not consider these commodities as very essential food items, thereby were unwilling to take the risk and going to procure these from distance places, since such meat and meat products are generally available only in certain allocated places. It is further known that the consumers of India largely prefer to have freshly cut chicken and, therefore, about 90% of broiler sales in the country are confined to unorganized retail outlets. The huddles in the unrestricted inter-state movement of the product in several parts of the country have been also a major bottleneck.

While the closure of restaurants including those of the fast-food restaurants or quick-service restaurants further had greatly impacted the demand, the disruption of transport chains, perishability of the produce, and closure of several wholesale markets and malls in the cities affected the supply chains. Lack of sufficient storage facilities, as in case of eggs, in large layer farms and also cold-chain facilities led to forced disposal of the produce at a through-away price. Several shocking incidents such as burying thousands of live birds to avoid the spread of COVID-19; killing and burning the birds in masses; and just giving them for free due to decline in sales have been reported from different places of the country. As per the estimate, the COVID-19 scare and lockdown impacted 10 lakh broiler poultry farmers and 2 lakh layer farmers, and by the end of April 2020, the losses due to the same were estimated at Rs. 27,000 crores [12]. As per the information available, in certain places, the price of live poultry birds tanked to as low as Rs 10-30/kg, especially during 1^st^ week of lockdown when the poultry farmers were not prepared for such unique situation of disconnection with the markets [12]. With the subsequent relaxation of transport chains, although there has been a certain recovery in the demand front, there remains also a great degree of supply-demand gaps, affecting largely the industrial poultry sector. Further, it is expected that even after the lifting of lockdown, the increase in demand for chicken and eggs may happen only in a gradual manner.

Several of the small poultry farms even though planned to continue the grow-out farming activities with a hope to have remunerative price from the sale of the products on a later period, most of them did not have adequate access to the feed supply from the local agents or feed plants situated in distant places. The backyard poultry sector, which was largely confined to the small and marginal farmers and landless laborers, probably have been equally affected due to the non-availability/non-accessibility of the nearby village markets and having limited access to the adjacent smaller townships. The poultry hatcheries have also been highly affected in these days due to almost no demand for chicks from the growers. During the initial days of lockdown, the hatcheries were even seen in throwing the incubated eggs and freshly hatched chicks. Although the government has permitted for the continuance of farming activities, hatchery operation and other activities associated with the supply and market value chains, there remains a crisis of non-availability of adequate labor due to the non-accessibility of adequate public transport and general fear of COVID-19 infection among the daily-wage labors.

## Impact on Nutrition and Health Care of Livestock and Poultry

Inadequate availability of critical inputs such as feed and fodder, at least during the 1^st^ phase of lockdown, significantly affected the growth and production of the rearing animals, leading to substantial economic loss. Due to the shutting down of the feed plants, availability of the animal feed in most of the places was difficult. With no proper access to the feed supply, the dairy farmers in the initial period had to compromise the feeding of their cattle and buffaloes largely with the available dry crop residues and brans. However, with the revival of supply system from the feed plants to the distributors which increases the availability of feed at the local markets, and also with the availability of the adequate transport facilities, the feed access by the livestock and poultry farmers has not remained a constraint in most of the places in recent times.

Limited access to the veterinary dispensary or even veterinarians and also problems of transportation of animals to the polyclinic at the time of need led to a high level of morbidity and at times death of the animals. This limited health care in long run would greatly impact the reproductive efficiency and productivity of the animals. The routine vaccination program carried out by the Governments for Foot and Mouth Disease (FMD) at 6-monthly intervals, and hemorrhagic septicemia annually for both cattle and buffalos has not been undertaken in any of the states during this period, which may have a high ramification in controlling the disease outbreaks in coming months. Similarly, the halt of a surveillance program for the important livestock and poultry diseases undertaken under the ICAR-NIVEDI, Bengaluru, and also collection of serum samples for serro-surveillance would cause a major setback towards disease forecasting.

## Impact on Labor Availability

A large share of livestock production sector being dominated by the small/marginal farmers and landless laborers, it is expected that the non-availability of labor probably was not a serious issue, only except in larger dairy units which employ a huge number of the labor force. However, the non-availability of adequate labor for the organized poultry farms, hatcheries, feed plants, and also dairy processing units had been a constraint, especially during 1^st^ phase of lockdown, which, however, being resolved gradually. Livestock production involving various physical activities and disruption in the marketing of livestock and poultry products was affected by a labor shortage. Surprisingly, the emergency has seen a reverse migration of labor force from the urban to rural areas. In this context, it would be relevant to understand the socio-psychological aspects of these migrant laborers and provide necessary help and support for maintenance of their livelihood at the place of destination.

## Accomplishments and Strategies for Revival

To bring back the sector into normalcy, both the Union Government and State Government have taken several strategies, including declaring supply of livestock and poultry products under essential services, ensuring huddle-free interstate transport of livestock and poultry produce, facilitating the procurement of higher amount of milk through dairy cooperatives, providing several advisories for stakeholders associated with the sector through different organizations, and so on. The Ministry of Fisheries, Animal Husbandry and Dairying, and Government of India are reported to have written letters to the Chief Secretaries of all states and UTs underlining the importance of formulated feed and feed ingredients for the production of livestock and poultry birds and seeking necessary facilitation for their assured supply. To ensure safety and hygiene among the stakeholders, NDDB also has issued several guidelines for the milk producers and dairy farmers, village milk collection centers/BMC/chilling centers, dairy processing units, cattle feed plants, deport/branch offices/wholesale distributors/retailers, etc. [8]. It is also reported that the 19 Research Institutes under the ICAR have developed several advisories for different clientele groups, and also extending relevant technical guidance to the farmers and also industries, besides involvement of some of the institutes such as ICAR-NIHSAD, Bhopal; ICAR-IVRI, Izatnagar; ICAR-NRC on Equine, and Hissar in the analysis of COVID-19 samples in human.

Despite all facilitations, the associated industries such as feed plants, vaccine manufacturing units, and semen cryobanks have not been functioning at their fullest capacity, largely due to the shortage of labor and reduced demand, therefore, requiring urgent attention. As compound cattle feed is a crucial input for milk production, NDDB has extended all possible formulation support to all cattle feed plants across the country to smoothen the production process as per its Press Release on April 23, 2020 [8]. Although opening up of the market gradually has increased the demand for the produce and relevant value-added products, the experience gained on e-marketing needs greater encouragements and facilitations.

Poultry being one of the cheapest source animal proteins with its round the year availability all across the country, the consumption of poultry meat will increase substantially once the people start connecting to the market. With the clarification of no link of COVID-19 with chicken consumption by the government from time to time, the non-vegetarian population seems to have conviction now on the safety aspect of chicken consumption. With the lifting of all restriction by Government of India on every activity of poultry farming and associated production and supply value chains, it would be possible that the industry is revived and get back to the normalcy. The question that whether the industry can be able to get up on their own and start running has remained unanswered. In this case, the industry would seek a stimulus from the government or the industrial enterprises with a certain degree of financial or associated logistic support. On the long run, it is also expected that there would be a change in the mindset of the consumers toward larger acceptance to the chilled/frozen chicken and also other value-added products, bringing strong processing, and marketing value chains in coming years.

Looking at the future danger of disease outbreaks in the livestock, it would be necessary that immediate action is taken to continue the planned vaccination programs for the bovines and other animals as relevant. It would be also necessary that due scientific and technical backup is provided for initiation of the halted surveillance program and strengthening the capacity to tackle the threat of any transboundary disease outbreak in the country in years to come. It would be necessary that all stakeholders associated in the sector, including those of producers, workers engaged in the value chains and also the consumers are made aware about the personal hygiene and quality maintenance of the products through aggressive awareness campaign and training to minimize the chances of COVID-19 disease spread.

Since COVID-19 has led to reverse migration of labor, the local administration must engage these returned laborers in gainful employment. The presence of an additional member(s) in the household without any immediate additional income and contribution to food production may lead to a challenging situation for maintaining food and nutritional security at the household levels [14]. Therefore, it becomes necessary for the governments to ensure the availability of credit through the National/Cooperative banks with reduced or no interest rates, for those entrepreneurs/companies who propose to initiate or expand the activities of livestock and poultry farming or to meet their working capital requirements. Further, the grant of loans by the banks can be encouraged against the stock of long shelf-life products such as SMP, butter, and ghee [15]. About 100% interest subvention on all borrowing for 2 years can help in for further investment by the entrepreneurs and reviving the production pace by the farms. Incentivization of the entrepreneurs for setting-up relevant infrastructure facilities and operate such infrastructure, namely, poultry processing units, dairy processing plants, and cold storage by providing necessary credit support and subsidies would help in creating an untapped domestic market for the processed and value-added products on a long run.

At a time when the industry is plagued with the dearth of investment by the private sector, the recent launching of the two national programs, namely,, National Animal Disease Control Programme for FMD and Brucellosis with an outlay of Rs. 13,343 crores for ensuring 100% vaccination of cattle and farm animals, and creation of Animal Husbandry Infrastructure Development Fund with the allocation of Rs. 15,000 crores would serve as triggering factors for reviving growth pace of the livestock and poultry sector.

## Conclusion

The lockdown no doubt had a paramount significance toward prevention and risk mitigation from the deadly COVID-19 disease but had a significant adverse impact on the availability of the product from the livestock and poultry for the day-to-day consumption by the public at large and all those associated with the sector for their livelihood and/or income. The danger of the epidemic requiring social distancing and following several precautionary measures, and also the lockdown situation have taught each one of us several lessons to be better prepared for coping up with similar such situation in coming days and years. Although the impact perceived as on today gives an overall qualitative picture about the present gross scenario, a greater effort hitherto is necessary to take a holistic view on the impact on each of the sub-sectors and associated value chains through the collection of primary data all over the country and analyzing it through intellectual groups and apex organizations for arriving the quantitative figures. In this context, it is expected that the government would take all possible measures to combat the distressful situations through effective governance, necessary financial support, and creating a congenial environment for reviving the sector and ensuring the livelihood of the associated stakeholders. In this endeavor, the role of the private sector, NGOs, and even the common citizens would also be significant.

## Authors’ Contributions

JB, KV, HR contributed to the original draft and conception of the specific review. JB, KV contributed to the review and editing. HR supported in supervision. JB, KV and HR worked on the final approval of the version to be published. All authors read and approved the final manuscript.
